# Autophagy impairment in a mouse model of neuropathic pain

**DOI:** 10.1186/1744-8069-7-83

**Published:** 2011-10-24

**Authors:** Laura Berliocchi, Rossella Russo, Maria Maiarù, Alessandra Levato, Giacinto Bagetta, Maria Tiziana Corasaniti

**Affiliations:** 1 Department of Health Sciences, University "Magna Græcia" of Catanzaro, 88100 Catanzaro, Italy; 2 Department of Pharmacobiology and University Center for Adaptive Disorders and Headache (UCHAD), Section of Neuropharmacology of Normal and Pathological Neuronal Plasticity, University of Calabria, 87036 Arcavacata di Rende, Italy

**Keywords:** neuropathic pain, spinal nerve ligation, autophagy, LC3, Beclin 1

## Abstract

Autophagy is an intracellular membrane trafficking pathway controlling the delivery of cytoplasmic material to the lysosomes for degradation. It plays an important role in cell homeostasis in both normal settings and abnormal, stressful conditions. It is now recognised that an imbalance in the autophagic process can impact basal cell functions and this has recently been implicated in several human diseases, including neurodegeneration and cancer.

Here, we investigated the consequences of nerve injury on the autophagic process in a commonly used model of neuropathic pain. The expression and modulation of the main autophagic marker, the microtubule-associated protein 1 light chain 3 (LC3), was evaluated in the L4-L5 cord segment seven days after spinal nerve ligation (SNL). Levels of LC3-II, the autophagosome-associated LC3 form, were markedly higher in the spinal cord ipsilateral to the ligation side, appeared to correlate with the upregulation of the calcium channel subunit α2δ-1 and were not present in mice that underwent sham surgery. However, LC3-I and Beclin 1 expression were only slightly increased. On the contrary, SNL promoted the accumulation of the ubiquitin- and LC3-binding protein p62, which inversely correlates with autophagic activity, thus pointing to a block of autophagosome turnover.

Our data showed for the first time that basal autophagy is disrupted in a model of neuropathic pain.

## Background

Neuropathic pain is initiated or caused by a primary lesion or dysfunction in the nervous system, either peripherally or centrally [1]. It is now clear that pain shares a lot of similarities with other neurobiological processes such as learning and memory [2]. However, the participation of cellular and molecular mechanisms typical of conditions in which these processes are altered, i.e. in neurodegenerative diseases, has not received so far adequate attention in respect to the development and maintenance of pain states. For instance, the contribution of spinal neuronal cell death to neuropathic pain has been investigated in several animal models, but it remains still controversial. In fact, while apoptosis is reported to be responsible for the selective loss of GABAergic inhibition in the superficial dorsal horn of the spinal cord [3], it is questioned whether this cellular loss is necessary for the development of pain behaviour [4]. However, research so far has focused only on one mode of cell death, namely apoptosis, without considering other mechanisms leading to neurodegeneration or to neuronal dysfunction.

Here, we investigated whether degenerative mechanisms other than apoptosis may be modulated in a common experimental model of neuropathic pain. In particular, we focused on autophagy, a major intracellular degradation pathway lately implicated in several pathological conditions and brain diseases [5].

Autophagy is a common cytoprotective mechanism serving homeostatic functions such as cytoplasmic, protein and organelles turnover, and providing metabolic substrates when cell energetic demands are increased [6]. Basal autophagy is a highly regulated process and an imbalance in its fine tuning, both towards an increase or an inhibition, may be detrimental for cell survival. Cellular autophagic activity is usually low under basal conditions, but can be markedly upregulated by numerous stimuli. The most known inducer of autophagy is nutrient starvation, both in cultured cells and in intact organisms [7]. Autophagy can also be activated by stress stimuli such as hypoxia, energy depletion, endoplasmic reticulum stress and hormonal stimulation, by pharmacological agents like rapamycin, as well as in bacterial, viral, and parasitic infections [7]. Conversely, autophagy suppression is also often associated with a number of diseases. These include some forms of cancer, neurodegenerative disorders, infectious diseases, and inflammatory bowel disorders [5]. Also, a decline in autophagy function is a common feature of aging [8].

At least three forms of this process have been identified--chaperone-mediated autophagy, microautophagy, and macroautophagy--that differ with respect to their physiological functions and the mode of cargo delivery to the lysosome. Macroautophagy (usually referred to as autophagy) is the major form and consists in the initial formation of the isolation membrane or phagophore, which expands and closes around the cytoplasmic cargo to form a double-membrane autophagosome. This then fuses with a lysosome to form an autolysosome where the captured material is degraded together with the inner membrane [5]. A number of molecules take part to this dynamic and highly regulated process. Among these, autophagy-related (Atg) proteins are critical players. In particular, yeast Atg6, Atg8 and their mammalian homologues Beclin 1 and LC3, are required for the formation of the isolation membrane and the autophagosome [9].

When autophagosome formation is impaired, the subsequent accumulation of damaged organelles and protein aggregates interferes with cellular functions and may eventually lead to cell death. In the CNS, these detrimental effects are exacerbated by the non-proliferative nature of neuronal cells and by their specific architecture. In fact, the massive accumulation of defective organelles and/or protein aggregates resulting from autophagic failure may disturb axonal traffic and lead to enhanced neuronal toxicity [10].

Aim of this study was to investigate whether the autophagic process is altered in the spinal cord following spinal nerve injury. The expression of the main autophagic markers LC3 and Beclin 1 was investigated in the spinal dorsal horn of adult mice following spinal nerve ligation (SNL). SNL promoted the appearance of LC3-II, the autophagosome-associated LC3 form. However, LC3-I and Beclin 1 expression were not dramatically increased. In contrast, the ubiquitin- and LC3-binding protein p62 (also known as sequestosome-1/SQSTM1) was strongly up-regulated, thus pointing towards a block of autophagosomes turnover in this experimental model of neuropathic pain.

## Results

### Spinal nerve ligation induced a severe mechanical allodynia

**Figure 1:**
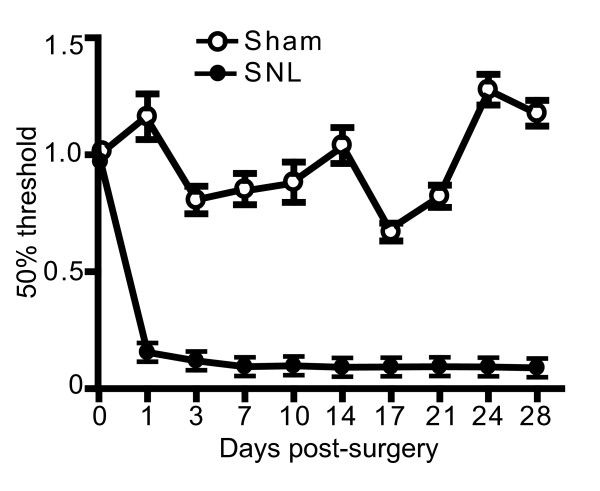
**Spinal nerve ligation induced a severe mechanical allodynia**. A severe and persistent mechanical hypersensitivity developed and maintained over time following SNL and was absent in animals that underwent sham surgery. Data were expressed as mean ± SEM of 50% of pain threshold and were normalized to the baseline of each animal (sham: n = 6; SNL: n = 10). For all time points, p < 0.001.

Ligation of the spinal nerve L5 produced an early onset and long lasting mechanical hypersensitivity (Figure 1), as previously described [11-13]. Tactile hypersensitivity determined by Von Frey filaments developed 1 day after SNL (n = 10) and was maintained for up to 28 days (Figure 1). The same marked decrease in mechanical thresholds was not observed in the sham group (n = 6; Figure 1).

### Spinal nerve ligation induced LC3 and Beclin 1 modulation

LC3 and Beclin 1 are two key proteins for autophagy function. Beclin 1 plays a role in the initiation step and is essential for the formation of the autophagosome [14]. LC3 exists in two forms. LC3-I is the cytosolic unconjugated form, which upon conjugation to phosphatidylethanolamine is recruited to the autophagosomal membrane. Due to this lipidation, LC3-I exhibits a greater electrophoretic mobility and is indicated as LC3-II. Since LC3-II levels are indicative of the rate of autophagomes formation, one of the most reliable methods to identify autophagy is the analysis of LC3 expression and LC3-II formation by Western blotting [14].

We investigated LC3 and Beclin 1 expression by Western blot in the cord of mice that underwent either SNL or sham surgery. Seven days after ligation, the expression of these autophagic markers was analysed in the L4-L5 portion of the spinal cord controlateral (C) and ipsilateral (I) to the ligation.

**Figure 2:**
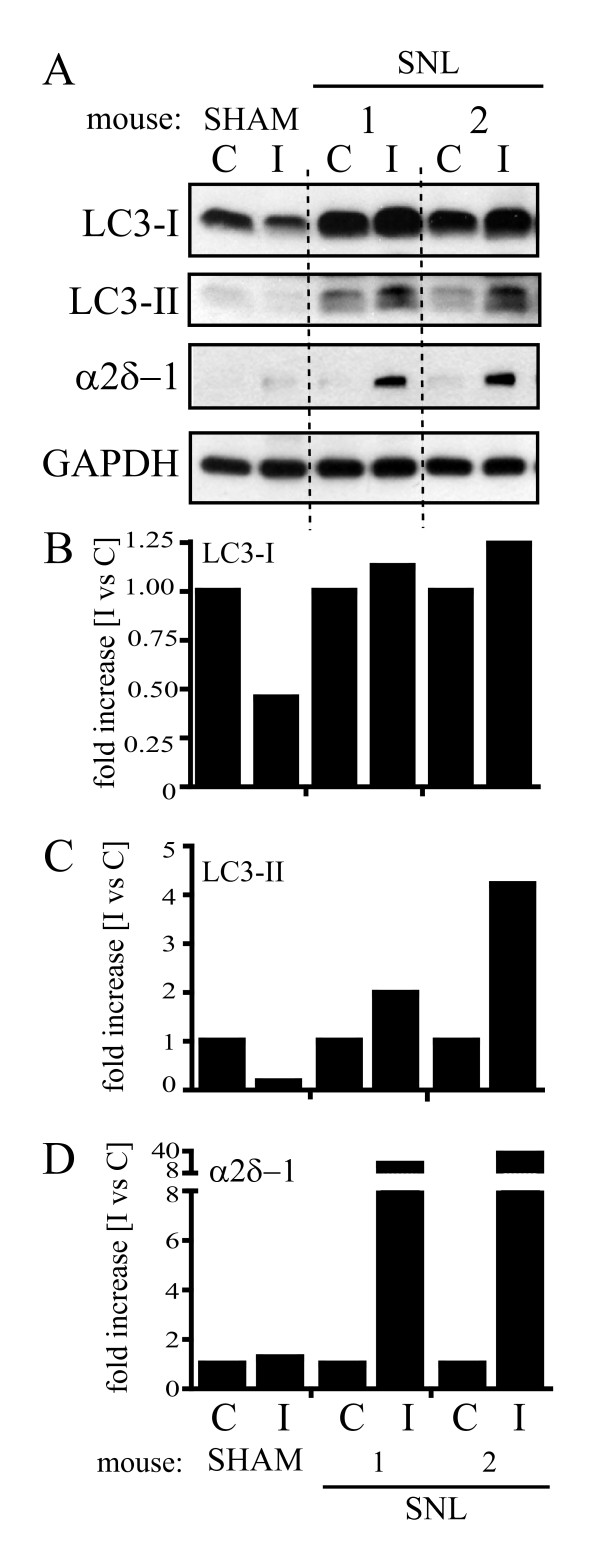
**LC3 expression in the spinal cord following spinal nerve ligation**. (A) LC3 expression was analyzed by Western blot in the hemi-cord contralateral (C) and ipsilateral (I) to the side of ligation, 7 days after surgery. SNL animals showed higher LC3-I expression than sham animals (A). A clear trend towards an increased LC3-I expression in the ipsilateral (I) compared to the contralateral (C) side, together with the appearance of LC3-II, was observed after ligation (A) and confirmed by densitometric analysis (B and C). The slight increase in LC3-I levels and the apparent formation of LC3-II well correlated with α2δ-1 upregulation (A and D). Signals from each band were normalized towards the corresponding GADPH signal. One representative Western blot is shown. Sham: n = 5, SNL: n = 6.

LC3-I levels were higher in the mice that underwent L5 ligation than in sham mice (Figure 2A). Also, the injured side (I) of SNL animals showed a slight increase in LC3-I levels in comparison to the controlateral side (Figure 2A and 2B). Moreover, the appearance of LC3-II was evident in the injured side of SNL animals but absent in sham mice (Figure 2A and 2C).

**Figure 3:**
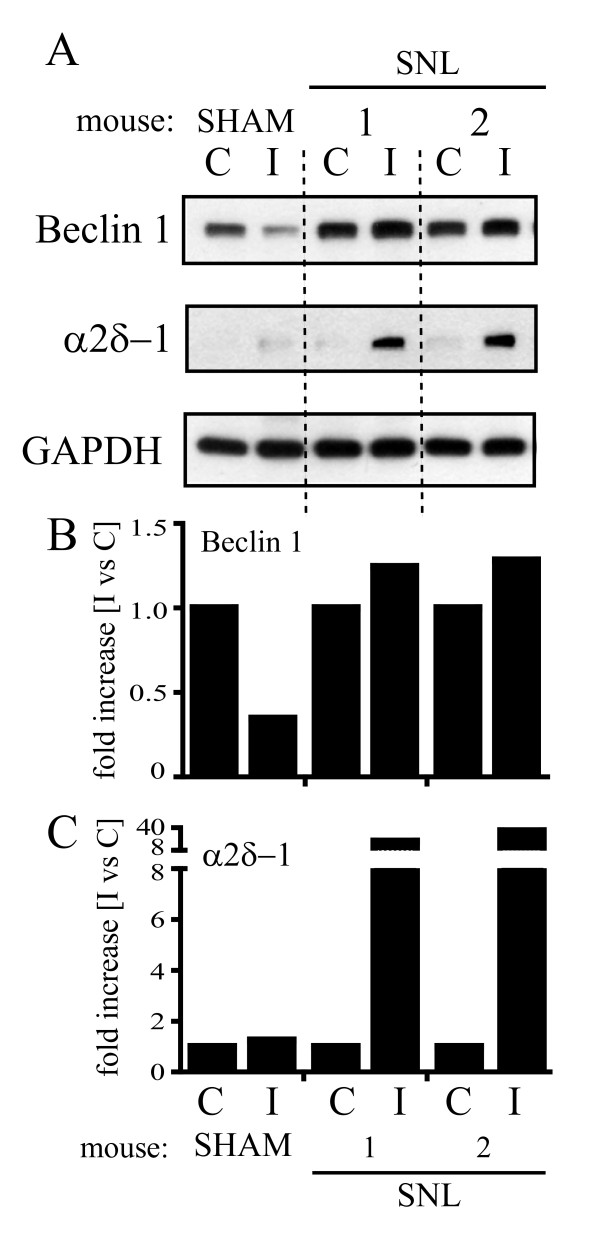
**Beclin 1 expression in the spinal cord following spinal nerve ligation**. (A) Beclin 1 expression was analyzed by Western blot in the hemi-cord contralateral (C) and ipsilateral (I) to the side of ligation, 7 days after surgery. SNL animals showed higher Beclin 1 expression than sham animals (A). A clear trend towards an increase in Beclin 1 expression in the ipsilateral (I) compared to the contralateral (C) side was observed after ligation (A) and confirmed by densitometric analysis (B). The slight increase in Beclin 1 expression well correlated with α2δ-1 upregulation (A and C). Signals from each band were normalized towards the corresponding GAPDH signal. One representative Western blot is shown. Sham: n = 5, SNL: n = 6.

Also Beclin 1 expression was higher in animals that underwent L5 ligation than in sham mice (Figure 3A), and a trend towards an increase was observed in the ipsilateral in comparison to the controlateral side 7 days after SNL (Figure 3A and 3B).

Given the variability in tissue sampling due to the small portion of cord taken and to ensure that the variations observed were related to the pain condition, we used the calcium channel subunit α2δ-1 as a reference for the specific lumbar segment and as a marker for the neuropathic pain state. Ipsilateral increase of the α2δ-1 subunit has, indeed, been reported in the dorsal horn after SNL. This up-regulation starts at about 7 days post surgery and is confined to the cord segment relative to the injured nerve [15,16].

Variations in both Beclin 1 (Figure 3A) and LC3-I levels (Figure 2A), as well as LC3-II formation (Figure 2A) well correlated with α2δ-1 upregulation in the injured side of SNL animals (Figure 2A and 2D, Figure 3A and 3C).

**Figure 4:**
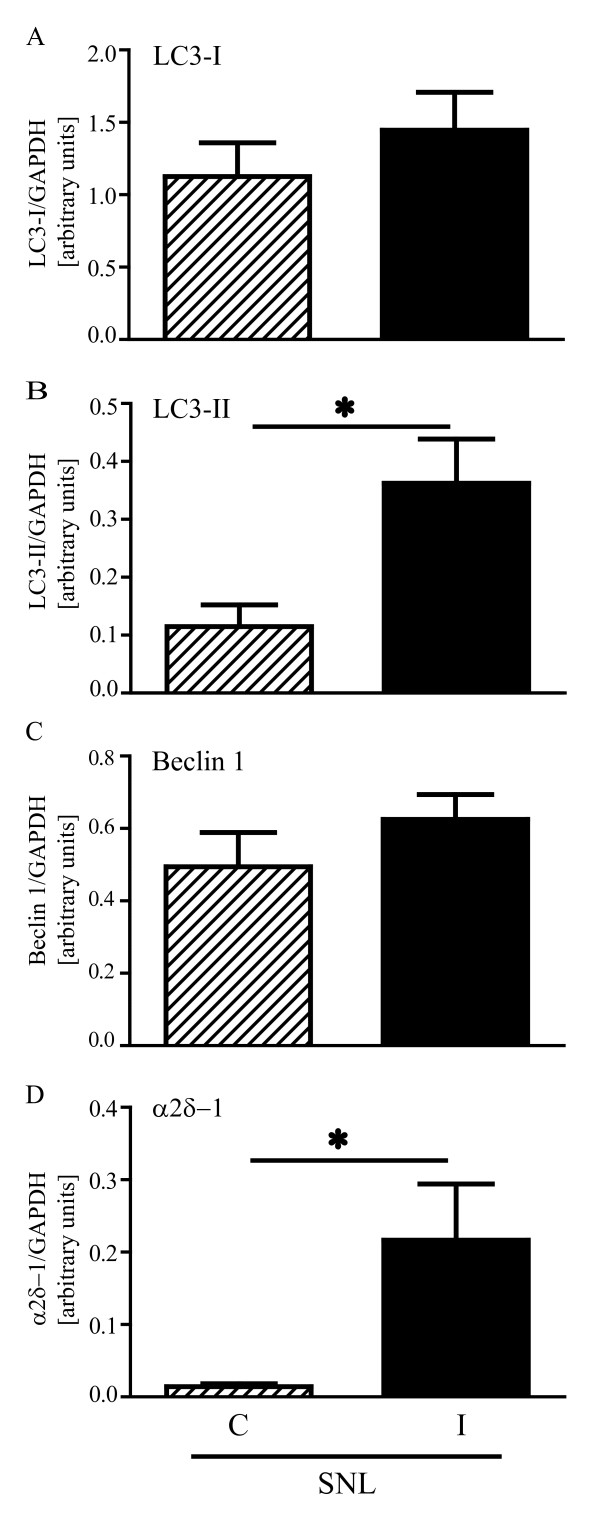
**Statistical analysis**. Quantitative analysis of Western blots by densitometry confirmed a trend towards an increase in LC3-I (A) and Beclin 1 (C) expression in the ipsilateral (I) compared to the controlateral (C) side of SNL animals 7 days after surgery. In the same animals, this was accompanied by a marked increase in LC3-II levels (B) and associated to strong α2δ-1 upregulation (D). For LC3-I, LC3-II, Beclin 1 and α2δ-1: n = 5. Signals from each band were normalized towards the corresponding GAPDH signal. Values were expressed as mean ± SEM, * p < 0.05.

While LC3-I and Beclin 1 showed only a slight increase following spinal nerve ligation, LC3-II formation and α2δ-1 upregulation were statistically significant as shown by statistical analysis of the densitometry data from 5 SNL mice (*p < 0.05; Figure 4).

### p62 upregulation following spinal nerve ligation

LC3 is regarded as a reliable biochemical indicator of autophagic activity, provided that the autophagy pathway is fully functioning. In fact, LC3-II accumulation may also be a consequence of defective autophagosome clearance rather than increased autophagy.

The ubiquitin-binding protein p62/SQSTM1 is an autophagy substrate, which upon direct binding to LC3 incorporates into autophagosomes and is efficiently degraded by autophagy [17,18]. Thus, when autophagy is blocked, p62 accumulation occurs [19]. To assess the hypothesis that LC3-II increase was a consequence of defective autophagosome clearance, we evaluated p62 levels by Western blot in the cord of mice that underwent either SNL or sham surgery. The expression of this marker was analysed in the L4-L5 portion of the spinal cord contralateral (C) and ipsilateral (I) to the ligation, 7 days after injury.

**Figure 5:**
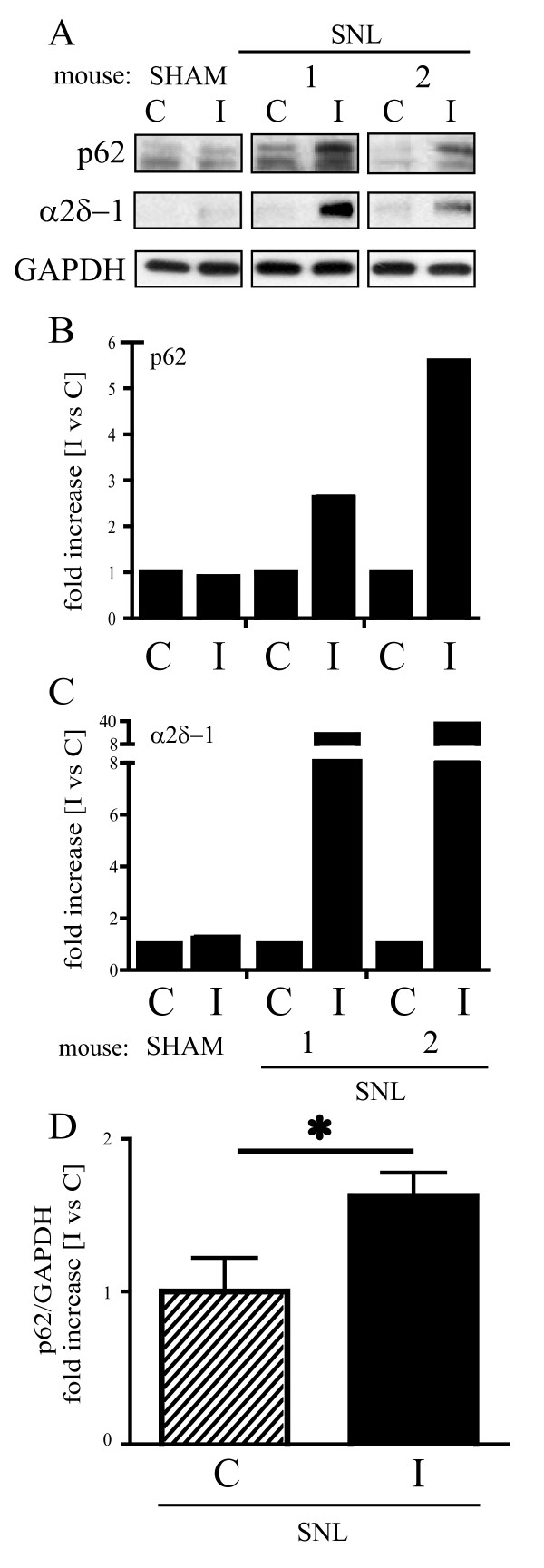
**p62 accumulation in the spinal cord following spinal nerve ligation**. (A) p62 expression was analyzed by Western blot in the hemi-cord contralateral (C) and ipsilateral (I) to the side of ligation, 7 days after surgery. The minor band detected by the antibody together with the main upper band has been previously suggested as a p62 splicing variant or a partially cleaved product [19]. SNL animals showed higher p62 expression than sham animals (A). A clear trend towards an increased p62 expression in the ipsilateral (I) compared to the contralateral (C) side was observed after ligation (A) and confirmed by densitometric analysis (B). The increase in p62 levels correlated with α2δ-1 upregulation (A and C) and was confirmed by quantitative analysis (n = 5, D). Signals from each band were normalized towards the corresponding GAPDH signal. Values were expressed as mean ± SEM, * p < 0.05.

Samples from mice that underwent L5 ligation showed higher p62 levels than sham mice (Figure 5A and 5B). Also, p62 accumulation was marked in the injured side (I) of SNL animals in comparison to the controlateral side (C) and was absent in sham mice (Figure 5A and 5B). As for Beclin 1 and LC3, also p62 accumulation well correlated with α2δ-1 upregulation in the injured side of SNL animals and was statistically significant as shown by statistical analysis of the densitometry data from 5 SNL mice (*p < 0.05; Figure 5D).

## Discussion

Chronic pain is a debilitating condition affecting life quality and has dramatic economic impact on national health systems. At present, our ability to treat this condition is limited and there is an urgent need for more effective treatments. This goal can be achieved through a better understanding of the neurobiology of pain and its molecular mechanisms.

Here, we investigated the consequences of nerve injury on the autophagic process in the spinal cord of mice that underwent SNL. As previously described [13], a robust and persistent hypersensitivity to mechanical stimulation developed from the first day following SNL, lasting up to 28 days. Seven days after injury, a strong α2δ-1 up-regulation was detectable in the ipsilateral spinal cord, in line with what has been observed in the same and other similar neuropathic pain models [15,16]. At this time point, LC3-II was markedly increased in the L4-L5 spinal cord segment ipsilateral to injury side in mice that underwent SNL, but it was absent in sham mice (Figure 2A and 2C). LC3-II formation was paralleled by a slight increase in LC3-I expression in the ipsilateral in comparison to the contralateral side (Figure 2A and 2B). LC3-II formation is regarded as a reliable biochemical evidence of autophagy since the amount of LC3-II usually correlates well with the number of autophagosomes [20]. However, the accumulation of autophagosomes is not always indicative of autophagy induction as it may represent either increased generation of autophagosomes and/or a block in autophagosomal maturation and in the completion of the autophagy pathway. We found that LC3-II formation was associated to a slightly higher expression of both LC3-I (Figure 2A and 2B) and Beclin 1 (Figure 3A and 3B), suggesting that autophagy was only mildly induced. On the contrary, LC3-II accumulation was paralleled by a drastic elevation of p62 levels (Figure 5A, B and 5D), strongly supporting the hypothesis of a blockade in the autophagic flux.

It is clear that disruption of the autophagic process in the CNS is deleterious, resulting in the accumulation of dysfunctional macromolecules and organelles. Atg5- or Atg7-deficient mice die within 1 day after birth [21,22], whereas CNS specific Atg5 or Atg7 knockouts are characterised by growth retardation, motor and behavioral deficits, extensive neuronal loss and die between 3 and 28 weeks after birth [23,24]. In these mice, the progression of autophagy disruption was shown to correlate with neurological dysfunction [24]. Moreover, the autophagic machinery is required for the remodelling of neuronal dendrites and axons and hence for maintaining CNS plasticity [25]. In neurodegenerative diseases, the impairment of this last function has been suggested as the cause for the progressive anatomical and functional alterations of the CNS occurring when massive cell loss is still undetectable [25].

The molecular and cellular processes responsible for the long-lasting abnormal sensory activation underlying chronic pain are still poorly understood. However, it is now clear that structural and functional changes in the spinal dorsal horn result in alterations in sensory processing following nerve injury, and contribute to the development and maintenance of neuropathic pain states [26]. Although our data do not answer as to whether autophagy disruption is an epiphenomenon or whether it is a critical event in pain processing, they provide a novel observation and open a new line for further investigations.

Basal and optimally induced levels of autophagy are particularly critical for neurons. Here, impaired autophagy can be deleterious not only by failing to provide energy for essential cell functions but also by allowing accumulation of damaged cellular components and dysfunctional proteins, as well as impairing receptors trafficking and degradation [27], thus affecting neuronal activity. Interestingly, a key regulator of autophagy is the growth regulating kinase mTOR. It has been speculated that, in the adult brain, the regulation at synapses of both autophagy and protein synthesis by mTOR activity is critical for different aspects of synaptic plasticity and that mTOR might act as a critical switch point that suppresses autophagy during synaptic strengthening (LTP) and induces it during LTD [27]. Recently, the mTOR signalling pathway has been implicated in the regulation of pain sensitivity [28]. Also, spinal mTOR inhibition by rapamycin has been shown to profoundly reduce pain sensitivity in the spared nerve injury (SNI) model of chronic pain [29], as well as to reduce formalin-induced pain-related behaviour [30]. Although these effects have exclusively been ascribed to rapamycin ability to block protein synthesis, also induction of autophagy may be a consequence of mTOR inhibition [31]. Therefore, further experiments will be needed to identify the correlation between the mTOR pathway and the block of autophagy observed in our model.

Although stronger on the side of injury, an increase in the autophagic markers was detected also in the controlateral cord, when compared to sham animals (Figure 2A, 3A and 5A). This may reflect an effect also on non-neuronal cells. In the spinal cord, Beclin 1 and LC3 upregulation have been recently reported after hemi-transection in both neurons and glial cells [32,33]. Although our data support the occurrence of a block in autophagosome processing, it is not clear at what late "digestive" step of the autophagy pathway this may occur. Autophagy dysfunction associated with autophagosomes accumulation in neurons has been linked to impaired vesicular transport [34], defective fusion of autophagosomes with lysosomes [35], or reduced lysosomal activity [36]. In particular, the actions of one or more cathepsins is essential for degrading autophagosome cargoes as shown by the autophagy failure and progressive neurodegeneration observed in cathepsin D as well as cathepsin B and L knockout mice [37]. In the light of the growing awareness on the role of glia in pain processing, further experiments are needed to investigate the role of autophagy in the different cell types.

In conclusion, our data showed that autophagy is disrupted in the spinal cord of mice that underwent SNL and suggest that accumulation of autophagy markers is likely to result from a block in completion of basal autophagy rather than up-regulation of the pathway.

To our knowledge, this is the first work reporting an impairment of spinal autophagy in a model of neuropathic pain and provides a platform for future studies on the role of this degradative pathway in pain processing.

## Material and methods

### Animals

Male C57BL/6 mice (20-22 g) (Charles River, Italy) were housed in temperature (22° C) and humidity (65%) controlled conditions and subjected to a 12-hour light/dark cycle (lights on at 8:00am), with free access to water and pelleted food (VRF1; Charles River, Italy). All the experimental protocols were in accordance to the guidelines of the Italian Ministry of Health for animal care (D.M. 116/1992).

### Surgery

Under 2% isoflurane anaesthesia, a midline incision was made in the skin of the back at the L_2_-S_2 _levels and the left paraspinal muscles separated from the spinal processes at the L_4_-S_1 _levels. The L6 transverse process was carefully removed and the left L5 spinal nerve was isolated and tightly ligated with 6-0 silk thread [11]. Complete hemostasis was confirmed and the wound was sutured.

The surgical procedure for sham group was identical to SNL group, except that the spinal nerve was not ligated. After surgery, foot posture and general mice behaviour were monitored throughout the postoperative period. Behavioural testing was carried out over a 4 weeks period.

### Behavioural test

Mice were acclimatized in the test chambers for at least 1 h until the exploratory activity had ceased. The behavioural tests were performed twice daily 3 and 5 days before surgery (baseline) and then 1, 3, 7, 10, 14, 17, 21, 24 and 28 days after spinal nerve ligation. Mechanical sensitivity was evaluated by Von Frey test [11,13]. The threshold was determined by using the up-down method as previously described [13,38] and data were expressed as mean of the 50% pain threshold ± SEM.

### Western blotting analysis

Mice were killed 7 days after surgery and the spinal lumbar segment (L4-L5) rapidly removed. The ipsi- and contra-lateral sides of each spinal cord were dissected, snap-frozen and stored at -80°C until further processing. For protein extraction, each single hemi-cord segment was homogenized in ice-cold lysis buffer (50 mM Tris-HCl (pH 8.0), 150 mM NaCl, 1 mM EDTA, 0,1% SDS, 1% IGEPAL, 0,5% Na-deoxycholate) in the presence of protease inhibitors (cod. P8349; Sigma-Aldrich, Milan, Italy) and incubated on ice for 40 min. Samples were then centrifuged at 14,000 × g for 15 min at 4 °C. Total protein content was determined in the supernatants by the Bio-Rad DC Protein Assay Kit (Bio-Rad Laboratories, Milan, Italy). For Western blot analysis, equal amount of total proteins were separated by sodium dodecyl-sulfate polyacrylamide gel electrophoresis (SDS-PAGE; 15%) and transferred onto PVDF membranes (Immobilon-P, Sigma-Aldrich, Milan, Italy). After blocking for 1 hour at room temperature in Tris-buffered saline containing 0.05% Tween 20 (TBST) and 5% non-fat milk, the membranes were incubated overnight at 4°C with the primary antibody directed against the protein of interest. After several washes, an appropriate HRP-conjugated secondary antibody (goat IgG; Pierce Biotechnology, Rockford, IL, USA) was applied for 1 hour at room temperature. Peroxidase activity was visualised using the ECL Western Blotting Detection kit (ECL, Amersham Biosciences, GE Healthcare, Milan, Italy) and X-ray films (Hyperfilm ECL, Amersham Bioscience). Signal intensity was measured using ImageJ software (NIH, Bethesda, MD, USA). For quantitative analysis, the Beclin 1, LC3-I, LC3-II, p62 and α2δ-1 signals of each sample were normalized versus the corresponding GAPDH signal. Changes in signal intensity were then expressed as fold increase of the ipsilateral versus the contralateral side for each individual animal.

The following primary antibodies and dilutions were used: anti-LC3 1:1000 (cod. PD036; MBL, Japan), anti-Beclin 1 1:4000 (cod. PD017; MBL, Japan), anti-p62/SQSTM1 1:1000 (cod. PM045; MBL, Japan), anti-α2δ-1 1:1000 (cod. D219; Sigma-Aldrich, Milan, Italy), anti-GAPDH 1:40000 (Applied Biosystem, Carlsband, CA, USA).

### Statistical analysis

Data were presented as mean ± SEM and statistically assessed either by two-way ANOVA (behavioral test) or by Student's t-test (Western blots). A value of p ≤ 0.05 was considered statistically significant. Calculations were done using GraphPad Prism (GraphPad Software, Inc).

## List of abbreviations

Atg: autophagy-related genes; CNS: central nervous system; LC3: microtubule-associated protein 1 light chain 3; mTOR: mammalian target of rapamycin; LTP: long-term potentiation; LTD: long-term depression; SNI: spared nerve injury; SNL: spinal nerve ligation; SQSTM1: sequestosome-1

## Competing interests

The authors declare that they have no competing interests.

## Authors' contributions

LB designed the experiments, analysed the data and wrote the manuscript, RR participated in the design of the experiments and in the analysis of the data, MM carried out the Western blots, AL carried out the behavioural study, GB conceived the study, MTC supervised experiments and corrected the manuscript. All authors read and approved the final manuscript.
